# Understanding the use of CATI and web-based data collection methods during the pandemic among digitally challenged groups at FQHCs: data from the *All of Us* Research Program

**DOI:** 10.3389/fdgth.2024.1379290

**Published:** 2024-06-04

**Authors:** Soumya Kini, Kimberly Marie Cawi, Dave Duluk, Katrina Yamazaki, Matthew B. McQueen

**Affiliations:** ^1^ The MITRE Corporation, Mclean, VA, United States; ^2^ Moses/Weitzman Health System, Middletown, CT, United States

**Keywords:** participant recruitment, underrepresented in biomedical research, *All of Us* Research Program, health equity, digital readiness, virtual data collection, computer assisted telephone interviewing (CATI), pandemic

## Abstract

**Introduction:**

The *All of Us* Research Program (Program) is an ongoing epidemiologic cohort study focused on collecting lifestyle, health, socioeconomic, environmental, and biological data from 1 million US-based participants. The Program has a focus on enrolling populations that are underrepresented in biomedical research (UBR). Federally Qualified Health Centers (FQHCs) are a key recruitment stream of UBR participants. The Program is digital by design where participants complete surveys via web-based platform. As many FQHC participants are not digitally ready, recruitment and retention is a challenge, requiring high-touch methods. However, high-touch methods ceased as an option in March 2020 when the Program paused in-person activities because of the pandemic. In January 2021, the Program introduced Computer Assisted Telephone Interviewing (CATI) to help participants complete surveys remotely. This paper aims to understand the association between digital readiness and mode of survey completion (CATI vs. web-based platform) by participants at FQHCs.

**Methods:**

This study included 2,089 participants who completed one or more surveys via CATI and/or web-based platform between January 28, 2021 (when CATI was introduced) and January 27, 2022 (1 year since CATI introduction).

**Results and discussion:**

Results show that among the 700 not-digitally ready participants, 51% used CATI; and of the 1,053 digitally ready participants, 30% used CATI for completing retention surveys. The remaining 336 participants had “Unknown/Missing” digital readiness of which, 34% used CATI. CATI allowed survey completion over the phone with a trained staff member who entered responses on the participant's behalf. Regardless of participants' digital readiness, median time to complete retention surveys was longer with CATI compared to web. CATI resulted in fewer skipped responses than the web-based platform highlighting better data completeness. These findings demonstrate the effectiveness of using CATI for improving response rates in online surveys, especially among populations that are digitally challenged. Analyses provide insights for NIH, healthcare providers, and researchers on the adoption of virtual tools for data collection, telehealth, telemedicine, or patient portals by digitally challenged groups even when in-person assistance continues to remain as an option. It also provides insights on the investment of staff time and support required for virtual administration of tools for health data collection.

## Introduction

The *All of Us* Research Program (*All of Us* or Program) is an ongoing longitudinal data collection operated by the National Institutes of Health (NIH) to collect lifestyle, health, socioeconomic, environmental, and biological data from 1 million US-based participants (1). Diversity is a core tenet of the Program, with a special focus on enrolling populations that are underrepresented in biomedical research (UBR). The Program has defined specific UBR categories that include racial identity, age when consented to Program participation, biological sex at birth, sexual orientation, gender identity, income, educational attainment, access to care, disability and rurality. As part of the enrollment process to the Program, participants complete registration, consent agreements, online surveys and provide biospecimens (blood, urine, and/or saliva) and physical measurements at enrollment sites (1). Once enrolled, they continue active involvement in the program through completion of online retention surveys and other data collection activities (2). The Program relies on Healthcare Provider Organizations, including Federally Qualified Health Centers (FQHCs), as well as direct volunteers to achieve its recruitment and retention goals.

FQHCs are a key recruitment stream of UBR participants and are centrally coordinated and supported by The MITRE Corporation (MITRE) (3). FQHCs are community based and provide primary care and preventive services in medically underserved areas regardless of ability to pay (4). Over 90% of FQHC patients are low income, over 80% are publicly insured or uninsured, and the majority are members of racial and ethnic minority groups (5). In *All of Us*, most recruitment and retention activities are completed via web-based portal from a computer or a mobile device. Therefore, digital readiness plays a key role in the FQHC *All of Us* team's ability to enroll and retain participants in the Program. Previous research utilizing a pre-pandemic sample of 2,791 FQHC participants showed that digital readiness is associated with younger age, higher education and income, and gender identity, with females being more likely to be digitally ready (6). Digitally ready participants also had 27% higher odds of completing Program activities than those not digitally ready (6).

Given a significant overlap between participants who are not digitally ready and those with low income, who are less educated, and of increased age, recruitment and retention of FQHC participants to the Program is a major challenge and requires education, training, and high-touch methods involving in-person staff assistance (7). Adoption of high-touch methods and meeting participants where they are helps build the trust needed to enroll and retain UBR populations in research (7). Additionally, studies have found that participants aged over 60 years participating in public health research preferred to have higher involvement in social activities compared to those that did not consent to participate (8). This further shows the need for high-touch methods to build rapport with study participants. However, high-touch methods such as in-person staff assistance ceased as an option for *All of Us* participants in March 2020 when the Program paused all in-person activities at all enrollment sites due to the pandemic to develop safety protocols and modified workflows for in-person activity (9).

Similar to *All of Us*, during the restrictive phases of the pandemic, many research programs adapted their survey data collection to virtual methods involving one-way or two-way communication strategies (10). One-way strategies included creation of videos for raising awareness, as well as instructions for online survey completion (10). Two-way communication strategies included using video calls such as through Zoom, Skype, or GoToMeeting (10). However, adoption of these methods, while successful, came with challenges and limitations. Use of digital platforms relied on the individuals having access, funds, and ability to use the technology and the internet, as well as the digital literacy to navigate through these processes (10). An alternative to video calls was the use of phone calls that were especially attractive to individuals with low digital literacy, even though phone calls were less natural than face-to-face interactions (11). Phone calls were also valuable in maintaining and developing relationships with study participants (12). When conducting surveys via mobile phones and online platforms, non-response rates tend to be higher among certain demographics, such as rural and elderly populations (13). Additionally, issues like distrust of unknown phone numbers, poor network coverage, and literacy levels affect participation rates. Online surveys, while capable of achieving high participation, tend to overrepresent higher-income, urban populations with greater access to smartphones and the internet. Further research is needed to address these challenges and maximize the effectiveness of virtual data collection methods (13).

In January 2021, the program introduced Computer Assisted Telephone Interviewing (CATI) as a method to help participants complete surveys remotely to minimize the impact of the pandemic on survey completion (14). CATI is a telephone survey methodology in which a trained interviewer follows a script to collect answers from participants. As the participant answers questions these responses are entered by staff into a database (15). CATI continues to be offered as a virtual option for participants for survey completion even after the Program has fully resumed their in-person activities, making this exploration relevant beyond the pandemic.

This paper aims to understand the association between digital readiness and mode of survey completion (CATI vs. Web-based platform) by participants who are patients at FQHCs. Analyses contained in this paper provide insights for NIH, healthcare providers, and researchers on the adoption of virtual data collection tools such as CATI for longitudinal data collection and studies, telehealth, telemedicine, or patient portals by digitally challenged groups when high touch methods such as in-person staff assistance continue to remain as an option. It also provides insights on the investment of staff time and support required to conduct the virtual administration.

## Methods

The analyses utilize data on adult FQHC patients who are *All of Us* participants collected by the following seven FQHCs from across the country that reflect the diversity of the United States: Community Health Center, Inc. (CHCI) located in Connecticut; Cherokee Health Systems (CHS) located in Tennessee; Cooperative Health (COOP) located in South Carolina; Jackson-Hinds Comprehensive Health Center (JHCHC) located in Mississippi; Sun River Health (SRH) located in New York, San Ysidro Health (SYH) located in California and Waianae Coast Comprehensive Health Center (WCCHC) in Hawaii. Data across all seven FQHCs were combined and analyzed. In addition, a deep-dive on participants from CHCI was included as a case-study.

The data collection methods were performed in accordance with relevant guidelines and regulations and approved by *All of Us* Research Program Institutional Review Board (IRB00010472). The participants included in this paper have provided consent to having their data used for research. Variables in the dataset are described in the sections below.

### Participant retention surveys

In *All of Us*, retained participants complete follow-up surveys at least once every 18 months after their enrollment. In this context, retention provides a measure for the ability of the FQHCs to sustain engagement with participants after recruitment to the Program. All retention activities, except submitting biospecimens to the Biobank, are completed on a web-based portal when participants come in-person to the FQHCs or virtually from a computer or a mobile device.

Data for this study are from Program participants who completed at least one or more of the following retention surveys during the study time period of January 28, 2021 (date when CATI was introduced by the Program) to January 27, 2022 (1 year following CATI introduction).

• Social Determinants of Health (SDoH) survey: Where the participant lives, their social life, feelings of stress, experiences with discrimination, and other everyday life experiences.
• Healthcare Access and Utilization (HCAU) survey: Access to health insurance and utilization of healthcare resources.
• Personal and Family Health History (PFHH) survey: Personal and family medical history, including medical conditions of biological parents, grandparents, siblings, and offspring.
• COVID-19 Participant Experience (COPE) and/or Minute survey: COPE surveys focused on how the pandemic impacted physical and mental health, daily life, and communities. Minute surveys focused on perceptions and experiences related to COVID-19 vaccines. Multiple versions of the COPE and Minute surveys were administered throughout the pandemic. Unlike the other three surveys above, a participant could take one or more versions of these surveys. However, only surveys completed during the study time period were included in the analysis, which included one COPE and four Minute surveys.

### CATI and web-based platforms

CATI was embedded into the existing infrastructure of the Program to minimize implementation cost and reach participants and program staff throughout the nation (15). The Participant Portal, already in use by the Program, was used for participant account access. The Program Management Toolkit (PMT) was used for Program staff access and data entry. To allow for staff-administered surveys via CATI, the Participant Portal was made accessible to staff to help participants navigate through the portal upon permission from the participant. Staff entered data collected from the CATI session in PMT. Meta data were collected in PMT, including the time to complete retention surveys administered via CATI or completed by the participant via the Web-based platform. The goal of CATI was to increase participation and retention of participants by providing the choice to allow survey administration over the phone. CATI was considered as an optional mode of survey administration for participants who expressed need or interest in this method.

### Minimum common metrics data

Minimum Common Metrics (MCM) is an Institutional Review Board (IRB)-approved questionnaire collected by FQHCs for MITRE. It contains participant responses about enrollment experience, digital readiness, access to a fitness tracker, and level of FQHC staff assistance required to complete *All of Us* activities. Questions related to digital readiness and the level of FQHC staff assistance required to complete *All of Us* activities were used in this study and are described in the next two sections.

### Digital readiness data (MCM)

Digital readiness was defined by access to home-based or other internet-accessing devices (computers, tablets, mobile phones, and other devices) and comfort level using such devices (6). Responses to the following three multiple-choice MCM technology access questions were utilized to define digital readiness:

1. Do you have access to a computer, tablet, or mobile phone at home? (Choices: yes, intermittent, no, prefer not to answer)
2. Do you have access to the internet through Wi-Fi or mobile data at home? (Choices: yes, intermittent, no, prefer not to answer)
3. How comfortable are you using technology, such as navigating emails, answering survey questions, or navigating a patient account portal? (Choices: very comfortable, somewhat comfortable, neutral, somewhat uncomfortable, not at all comfortable, prefer not to answer)

### Level of FQHC staff assistance

The level of assistance required by the participant to complete each of the retention surveys was recorded by the FQHC staff as part of MCM data collection. The level of assistance included the following multiple-choice selections.

1. Facilitated on-site (Potential participant was guided by FQHC staff at the FQHC facility and helped the potential participant navigate the process and explain content/answer questions)
2. Assisted on-site (FQHC staff helped the potential participant navigate through the process with limited contextual questions only as requested, while at the FQHC facility)
3. Facilitated Virtual Appointment (Potential participant was guided by FQHC staff through CATI and helped the potential participant navigate the process and explain content/answer questions)
4. Assisted Virtual appointment (Potential participant was guided by FQHC staff through CATI, with limited contextual questions only as requested)
5. Independent On-Site (Potential participants completed the consent process on their own at the FQHC facility)
6. Independent Off-Site (Potential participants completed the consent process on their own somewhere other than the FQHC facility)

Analysis of FQHC staff assistance focused on CHCI participants. Focusing on one specific FQHC allowed for a more focused analysis and minimized the potential variation between FQHCs. Of the surveys listed, the level of CHCI staff assistance for the COPE survey was not included since CHCI did not collect this data.

### CHCI data

In addition to data described in the sections above, CHCI also collected data on perspectives and experiences of frontline staff engaged in research with vulnerable populations. Insights, challenges, and successes in facilitating the surveys in person and virtually were captured during group discussions in team meetings. These meetings were organized to facilitate open dialogue among staff members, enabling the identification of common themes and diverse perspectives. The team meetings were conducted weekly to discuss ongoing projects, participate in professional development activities and engage in discussions related to the *All of Us* participant journey. Attendees included the Principal Investigator, Program Manager and frontline staff. Notes were taken during these meetings by designated team members, capturing discussions, comments and observations pertaining to challenges encountered by *All of Us* participants, as well as from the frontline staff. Meeting notes were compiled and organized to ensure coherence and accessibility for analysis. Results were compiled by CHCI staff through review of the notes, generation of initial themes and generating a report of challenges associated with the use of digital technologies.

### Study population

The study population included 2,089 *All of Us* participants who completed at least one or more of the aforementioned retention surveys between January 28, 2021 (date when CATI was introduced by the Program) and January 27, 2022 (1 year following CATI introduction) and responded to questions on digital readiness that were asked by FQHC staff at the time of their enrollment. The digital readiness questions were first asked by FQHCs in June 2019 to newly enrolled participants.

### Analytical methods

Analyses in this paper were conducted using Python (Python Software Foundation. Python Language Reference, version 3.9.16 available at http://www.python.org) and Microsoft® Excel® (Microsoft 365 MSO version 2309 Build 16.0.16827.20166 64-bit). Exact number of participants in groups less than 20 were not shown to stay consistent with the Program data suppression levels to support data privacy. *All of Us* Data and Statistics Dissemination (DSD) exception request was granted.

### Analytical data set

Participants that completed one or more of the aforementioned retention surveys between January 28, 2021 and January 27, 2022 and responded to the three MCM questions during enrollment were used to create the analytical data set. Variables corresponding to digital readiness disposition, mode of survey completion, time to complete, level of FQHC staff assistance (CHCI participants only) were available, as described below:

• Digital Readiness Status from MCM Data (6)
  ○ Digitally Ready Participants: Participants who responded with a “Yes” or “Intermittent” to Questions 1 and 2, and “Very comfortable,” “Somewhat comfortable,” or “Neutral” to Question 3 were categorized as *digitally ready*.
  ○ Not-Digitally Ready Participants: Participants who responded with a “No” to Questions 1 or 2 or “Somewhat uncomfortable” or “Not at all comfortable” to Question 3 were categorized as *not digitally ready*.
  ○ Unknown/Missing: Participants who skipped or selected the “Prefer not to answer” or “Unknown” option to any of the three questions were categorized as “Unknown/Missing”.
• Mode of survey Completion via CATI and Web
  ○ CATI only: Participants who completed at least one survey using CATI during the study time period and did not complete a survey using the web-based platform,
  ○ Web only: Participants that completed at least one survey via the web-based platform and did not complete a survey using CATI during the study time period.
  ○ CATI and Web: Participants that completed at least one survey using CATI and at least one survey using the web-based platform during the study time period.
• Time to Complete
  ○ Time to complete is defined as the time in minutes from when a participant opened a survey until a survey was submitted via CATI and/or Web. Time to complete in minutes up to 1 h were used for SDoH, HCAU, and PFHH surveys. COPE/Minute surveys were designed to be very brief and under a minute, so they were not included in the analysis for time to complete.
• Level of FQHC Staff Assistance from MCM Data (CHCI participants only)
  ○ Assisted or Facilitated by CHCI staff: Responses to questions 1 and 2 were consolidated into one category for on-site participants that completed surveys via the web-based platform. Similarly, selections 3 and 4 were consolidated into one category for CATI participants and were confirmed by the actual CATI appointments scheduled by the participant.
  ○ Independently Completed by the Participant: Selections 5 and 6 were consolidated into one category for participants that completed surveys on their own, on-site or off-site, via the web-based platform.

### Survey data completeness

Survey data completeness was used as a proxy measure for data quality from the surveys administered via CATI and web-based platforms. The HCAU survey was used to study completeness of survey responses since the largest number of participants in the study population responded to this survey during the study time period. The survey has 59 questions, and responses to these questions were tallied into categories shown below. A free text question and two questions that were only applicable to female participants were excluded from completeness calculations. Responses were grouped into various categories as defined below. Number of skips, as defined below, was used to assess completeness.

• Skips: When a participant was presented with a question and did not provide a response
• Don't Know: When a participant responded “Don't know” to a question
• None: When a question was not presented to the participant due to branching and/or skip logic
• Completed: When a participant provided a response other than “Don't Know” to a question

### Statistical tests

A Chi-square test was performed to test for significant differences between digitally ready, not digitally ready, and unknown/missing groups on their mode of retention survey completion at the alpha = 0.05 significance level.

The Kruskal-Wallis (KW) test and the Dunn's *post hoc* test with a Bonferroni corrected rejection region were conducted for several combinations of time to complete groups in the analytical dataset using the SciPy python library and scikit-posthocs package respectively. The KW and Dunn's tests were used to determine if there was a significant difference in medians.

## Results and discussion

### Relationship between digital readiness and mode of survey completions

In the study population of 2,089 FQHC *All of Us* participants, there were 1,053 digitally ready participants (50%), 700 not-digitally ready participants (34%), and 336 participants with “Unknown/Missing” digital readiness (16%). Table 1 shows the relative proportion of participants that were digitally ready compared to those not digitally ready broken down by their mode of completing retention surveys during the study time period.

**Table 1:** Relationship between digital readiness and the mode of retention survey completion.

Mode of survey completion	Digitally ready participants	Not-digitally ready participants	Unknown/missing digital readiness
CATI only	315 (30%)	356 (51%)	113 (34%)
Web only	630 (60%)	254 (36%)	194 (58%)
CATI and Web	108 (10%)	90 (13%)	29 (9%)
Total	1,053	700	336

Table 1 results indicate that among the 700 not-digitally ready participants in the study population, 51% (*n* = 356) used CATI only; and of the 1,053 digitally ready participants, 30% (*n* = 315) used CATI only for completing retention surveys. Among the 1,053 digitally ready participants, 60% (*n* = 630) used Web only for retention surveys. CATI provided an opportunity for participants to complete a survey over the phone with a trained FQHC staff member who entered responses on behalf of the participant. Therefore, CATI was used at a higher rate among not-digitally ready participants, perhaps due to their limited access to technology and their comfort level with technology in completing the surveys on their own. Preferences in the mode of completion between digitally ready and not-digitally ready participants were significant at 0.05 level [*X*(2) (4, *N* = 2,089) = 103, *p* < 0.05].

### Level of assistance for CHCI participants

Table 2 provides the relative proportion of CHCI staff assistance provided to participants for retention survey completions via the web-based platform. Analysis shows that 62% of survey completions among not-digitally ready participants required assistance or facilitation by CHCI staff members compared to 26% for digitally ready participants. Conversely, 74% of survey completions were done independently by participants that were digitally ready, compared to 38% by not-digitally ready participants.

**Table 2:** Level of assistance provided by CHCI staff based on participants’ digital readiness for web completions.

Level of assistance	Survey completions by digitally ready participants	Survey completions by not-digitally ready participants
Assisted or facilitated by CHCI Staff	118 (26%)	183 (62%)
Independently completed by the participant	328 (74%)	113 (38%)
Total web	446	296

### Relationship between digital readiness and time to complete on CATI and web

This section examines the relationship between digital readiness and time to complete the retention surveys via CATI and Web-based platforms (Table 3).

**Table 3:** Relationship between digital readiness and median time to complete retention surveys via CATI and web among FQHC participants.

	Digitally ready	Not-digitally ready	*p*-value**
HCAU			
Web	5.8 (*N* = 352)	7.5 (*N* = 139)	<0.0001***
CATI	9.0 (*N* = 108)	9.9 (*N* = 123)	0.36
*p*-value**	<0.0001***	0.0001***	
SDOH			
Web	13.2 (*N* = 224)	19.1 (*N* = 64)	<0.0001***
CATI	19.2 (*N* = 23)	17.4 (*N* < 20*)	0.39
*p*-value**	0.0008***	0.48	
PFHH			
Web	8.6 (*N* = 81)	12.0 (*N* = 21)	0.36
CATI	14.5 (*N* < 20*)	20.5 (*N* < 20*)	0.99
*p*-value**	0.052	0.23	

Table does not include participants categorized with digital readiness as “Unknown/Missing” and/or time to complete was not recorded.

* Exact number of participants in groups less than 20 were not shown to stay consistent with the Program data suppression levels to support data privacy. *All of Us* Data and Statistics Dissemination (DSD) exception request was granted.

** Dunn's *post hoc* test with a Bonferroni corrected rejection region was used for the calculation of *p*-values.

***A significant difference in medians was detected at the .0125 level.

Results from Table 3 indicate that the median time to complete surveys was longer for not-digitally ready participants (0.9–6.0 min longer for not-digitally ready participants). For participants using CATI to complete a survey, the median time to complete was 2.4–8.5 min longer compared to those using the web-based platform to complete the same survey. The only exception was the SDoH survey, where the median time to complete for not-digitally ready participants was 1.8 min shorter than digitally ready participants (17.4 vs. 19.2 min). Similarly, for not-digitally ready participants, the median time to complete was 1.7 min shorter for those using CATI compared to web-based platform (17.4 vs. 19.1 min). Given the small sample sizes, especially for CATI participants that completed SDoH survey (23 digitally ready and <20 not-digitally ready participants), these results may not be generalizable. However, the median completion times in Table 3 are within the ranges calculated from 9,399 *All of Us* participants from 39 enrolling organizations that took SDoH, HCAU and PFHH surveys via CATI and Web between January 2021 and January 2022, with CATI taking longer to complete (14). The median completion times were 10.6, 21.4 and 18.6 min for HCAU, SDoH and PFHH surveys, respectively, for participants that completed the surveys via CATI. For participants that used the web-based platform, the median completion times were 5.8, 12.5 and 11.1 min for HCAU, SDoH and PFHH surveys, respectively.

HCAU was the only retention survey that contained sample sizes greater than 100 participants across all groups. This was because the HCAU survey was launched by *All of Us* in June 2018 and was available for participants to take throughout the study time-period. In comparison, the SDoH and PFHH surveys were launched in November 2021, and were therefore only available for a short time for participants within the study time-period, resulting in a smaller sample size for these surveys.

For the HCAU survey, median time to complete using the web-based platform was 5.8 and 7.5 min for digitally ready and not-digitally ready participants, respectively. When using CATI, the median time to complete was at least 32% higher at 9.0 and 9.9 min for digitally ready and not-digitally ready participants, respectively. The KW test detected at least one significant difference in medians for the HCAU survey at the 0.05 level. The median completion times between CATI and web for the HCAU survey were significantly different using the *post hoc* Dunn's test at the 0.0125 level with a Bonferroni correction applied. Given consistently larger sample sizes for all groups of interest, the HCAU survey was used for deep dive analyses in subsequent sections.

### Level of assistance for CHCI participants

Table 4 provides median time to complete for the HCAU survey by CHCI participants via CATI and web-based platforms. It also shows time to complete by level of CHCI staff assistance among digitally ready and not-digitally ready participants. Of those participants who independently completed the survey on the web, the median completion times for not-digitally ready participants (9.0 min) was longer than the digitally-ready participants (5.4 min). CHCI data did not satisfy test assumptions, therefore KW and Dunn's test results are not reported. No observable differences in median time to complete were seen in individuals that required some level of assistance completing on the web (9.4 min for digitally ready and 10.6 for not-digitally ready) or through CATI (11.2 min for digitally ready and 11.8 for not-digitally ready), which is consistent with the broader FQHC population shown in Table 3 for HCAU survey administered via CATI only (9.0 min for digitally ready and 9.9 for not-digitally ready). This may be attributed to the participants requiring clarification responding to questions about survey items. CHCI staff held weekly team meetings to discuss aspects of the participant journey and any challenges they experienced. In these meetings, CHCI staff routinely mentioned that they spent more time clearing up confusion on the questions than helping the participants navigate digital technologies. Additionally, CHCI staff shared the HCAU survey was one of the faster surveys to assist/facilitate compared to the SDoH survey which took a greater amount of time for the participants to complete.

**Table 4:** Median time (in minutes) to complete health care access and utilization survey by CHCI participants via web vs. CATI and the level of staff assistance provided.

Mode of completion	Level of assistance	Digitally ready participants	Not-digitally ready participants
Web only	Assisted or facilitated by CHCI staff	9.4 (*N* = 34)	10.6 (*N* = 60)
	Independently completed by the participant	5.4 (*N* = 75)	9.0 (*N* = 20)
CATI only	Assisted or facilitated by CHCI staff	11.2 (*N* < 20*)	11.8 (*N* < 20*)

* Exact number of participants in groups less than 20 were not shown to stay consistent with the program data suppression levels to support data privacy. *All of Us* data and statistics dissemination (DSD) exception request was granted.

### Relationship between digital readiness and completeness of data on CATI vs. web

**Figure 1:**
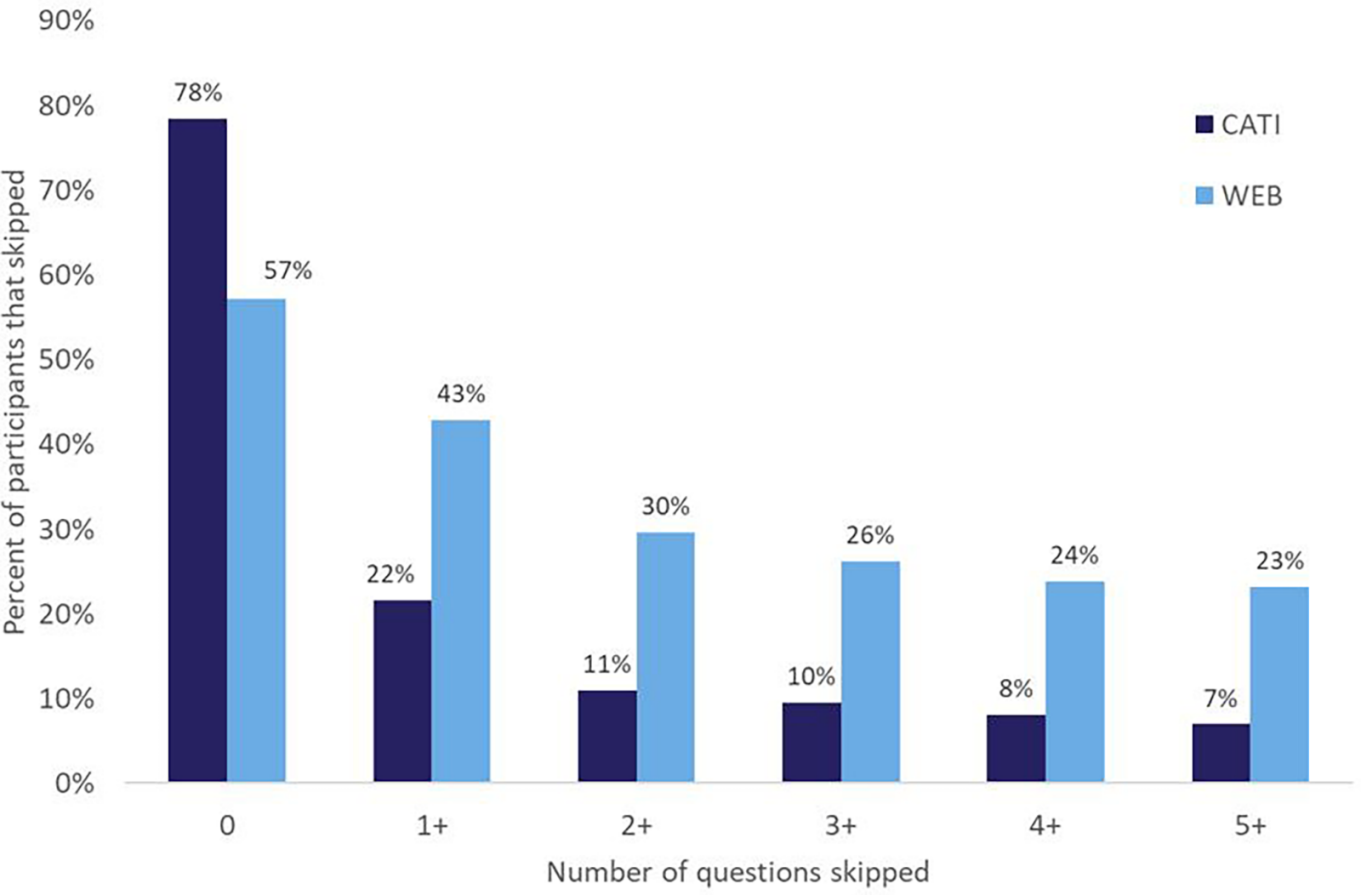
Percent of participants that skipped responding to questions via CATI and web for the HCAU survey.

This section examines whether using CATI resulted in more complete data for the HCAU survey. Because CATI involves a trained interviewer to collect answers from participants, the participant may be more encouraged to respond to a survey question than skipping it or providing a “Don’t Know” response. Figure 1^1^ provides a breakdown of the percent of participants that skipped responding to questions on the HCAU survey when asked and recorded by an interviewer via CATI vs. when the participant completed using the web-based platform.

Figure 1 shows that of the participants that used CATI to complete the HCAU survey, 78% responded to all questions without skipping. In comparison, 57% of participants that used the web-based platform responded to all questions without skipping. Of the 273 participants that used CATI, 59 participants (22%) skipped at least one question. Whereas about twice as many of the 614 participants that used the web-based platform (263 participants, 43%) skipped at least one question. About thrice as many participants that used the web-based platform skipped five or more questions (23%) compared to those that used CATI (7%). These findings demonstrate the effectiveness of using CATI for improving survey item response rates in online surveys, especially among population groups that are digitally challenged.

### Experiences among CHCI frontline staff

Discussions with frontline staff underscored a set of challenges for facilitating surveys among FQHC participants, notably a protracted survey duration attributable to language barriers, comprehension difficulties with survey questions, and participants' expressed desire to engage in substantive conversations about their responses. However, insights from these discussions also highlighted the rapport that is built between the CHCI staff member and participant. According to the CHCI staff, participants were more willing to answer all the questions because of the trust that was built between them during their dialogue. This may contribute to the decrease in number of skipped questions when surveys were completed with a frontline staff member as seen in Figure 1.

## Conclusions

The results presented provide valuable data-driven insights on the virtual survey completion experiences using CATI and wed-based platforms for digitally ready and not-digitally ready FQHC participants in *All of Us* during the pandemic. Findings suggest that CATI was more favored and utilized by not-digitally ready participants. About half of not-digitally ready FQHC participants used CATI—nearly twice as many as digitally ready participants. Data also supports the finding that survey completions via CATI need significantly more time than via the web-based platform. On one of the surveys, median time to complete was at least 32% higher using CATI compared to using the web-based platform. CATI resulted in fewer skipped responses than the web-based platform highlighting better data completeness. The improvement in data completeness could be attributed to longer time to complete surveys via CATI. On one of the surveys, about thrice as many participants that used the web-based platform skipped five or more questions compared to those that used CATI. These findings demonstrate the effectiveness of using CATI for improving response rates in online surveys, especially among population groups that are digitally challenged, or may benefit from increased investments in building trust. It also presents a case to consider digital readiness as an independent UBR category given the limitations and challenges, especially in studies that are digital by design. Broadly, this paper provides insights for NIH, healthcare providers, and researchers on the adoption of virtual tools such as CATI for longitudinal data collection and studies, telehealth, telemedicine, or patient portals by digitally challenged groups even when high touch methods such as in-person staff assistance continue to remain as an option.

## Limitations

The study was limited by the data that was already collected. The study sample sizes were limited by survey launch dates set by NIH. For example, the SDoH survey was launched in November 2021, which meant that only 3 months of SDoH survey data overlapped with the study population time period. This constrained opportunities for meaningful comparisons across multiple surveys for time to complete and survey completeness investigations. Future studies could leverage more data from the surveys that had sample size limitation in the current study (SDoH and PFHH) to provide insights for NIH and other stakeholders.

When analyzing completeness of data, measured as the number of skipped responses taken in a survey, one cannot make any assumptions on the honesty of the answers that were completed. For CATI, an honest answer may be given due to higher focus resulting from the interaction of a human asking the question, or an honest response may not be shared in the case of embarrassing or uncomfortable choices. Under-reporting socially undesirable responses results in social desirability bias (16). The accuracy of completed answers could not be evaluated in this study.

The MCM survey questions were not designed for this research paper. They were designed to understand general characteristics of the population that FQHCs enroll. MCM survey questions may also elicit social desirability bias. Participants were given the option to decline responding to the entire MCM survey or skip any of the questions. If a participant was uncomfortable responding to questions on technology devices and/or access, they may have chosen to skip.

Information on the device/technology (computer, mobile phone) used by the participant for the web-based surveys was not available. CATI was embedded into the existing infrastructure of the Program, with the only difference from the web implementation being the *All of Us* staff member entering the participant responses on their behalf rather than directly by the participant. Therefore, it is possible that in some situations, CATI presented a more user-friendly experience for participants who may have been limited by the device/technology available to them.

Finally, the results of this study must be viewed in connection with this longitudinal data collection effort, and may not generalize to other research efforts, which may have their own definition of digital readiness, retention, virtual administration tools, and may have a different study population. However, despite the limitations, the study provides valuable contributions by developing an objective and data-driven methodology to quantify the experiences of digitally ready vs. not-digitally ready groups on the virtual tools for completing surveys that are highly digital in nature, and in sustaining these groups for longitudinal data collection.

## Data Availability

The datasets generated during and/or analyzed in this study are available from the corresponding author on reasonable request, and subject to approval from NIH. Requests to access these datasets should be directed to skini@mitre.org.

## Footnotes

^1^*All of Us* Data and Statistics Dissemination (DSD) exception request was granted.
